# Thymalfasin combined with immune checkpoint inhibitors in the treatment of non-small cell lung cancer: A retrospective study on efficacy, safety, and immunological function

**DOI:** 10.12669/pjms.42.3.13385

**Published:** 2026-03

**Authors:** Peipei Wang, Xiaojing Zhang, Cheng Xiang, Guoying Li, Yajing Zhang

**Affiliations:** 1 Peipei Wang, Department of Oncology, Shijiazhuang People’s Hospital, Shijiazhuang 050000, Heibei, China; 2 Xiaojing Zhang, Department of Oncology, Shijiazhuang People’s Hospital, Shijiazhuang 050000, Heibei, China; 3 Cheng Xiang, Department of Oncology, Shijiazhuang People’s Hospital, Shijiazhuang 050000, Heibei, China; 4 Guoying Li, Department of Oncology, Shijiazhuang People’s Hospital, Shijiazhuang 050000, Heibei, China; 5 Yajing Zhang, Department of Oncology, Shijiazhuang People’s Hospital, Shijiazhuang 050000, Heibei, China

**Keywords:** Combination therapy, Efficacy, Immune checkpoint inhibitor, Immune function, Non-small cell lung cancer, Safety, Thymalfasin

## Abstract

**Objectives:**

To evaluate the efficacy, safety, and immunomodulatory effects of thymalfasin in combination with different immune checkpoint inhibitor (ICI)-based regimens in patients with advanced driver gene-negative non-small cell lung cancer(NSCLC).

**Methodology:**

A retrospective analysis was conducted on 120 patients with advanced NSCLC treated at Shijiazhuang People’s Hospital between January 2021 to December 2024. Patients were assigned to three groups: Group-A(*n=* 42), platinum-based doublet chemotherapy + ICIs + thymalfasin; Group-B(*n=* 44), single-agent chemotherapy + ICIs + thymalfasin; Group-C(*n=* 34), ICIs + thymalfasin. Assessed objective response rate(ORR), disease control rate(DCR), progression-free survival(PFS), overall survival (OS), adverse events(AEs), immunological indices, and quality of life score and 6-min walk distance [6MWD]).

**Results:**

ORR and DCR did not differ significantly among groups(*P>* 0.05, respectively). Median PFS and OS were significantly longer in chemotherapy-containing regimens(Groups-A and B) compared with Group-C(*P <*0.05, respectively), whereas no significant differences were observed between Groups-A and B. The incidence of AEs, including myelosuppression and gastrointestinal reactions, was comparable across groups(all *P>* 0.05). After treatment, all groups demonstrated significant improvements in immunological function(all *P<* 0.05), with Group-A showing the most pronounced increases in CD4^+^, IgG, and IgA levels compared with Group-C (*P<* 0.05, respectively). Improvements in FACT-L scores were most evident in Group-A (*P<* 0.05), and declines in 6MWD were smallest in this group(*P<* 0.05).

**Conclusion:**

Thymalfasin combined with ICIs and chemotherapy, particularly platinum-based doublet regimens, may significantly prolong survival, enhance immune function, and improve quality of life in patients with advanced NSCLC, without increasing treatment-related toxicity.

## INTRODUCTION

Lung cancer remains the leading cause of cancer-related mortality worldwide and in China. In 2022, China reported approximately 1.06 million new lung cancer cases and 733,300 deaths, posing a major public health burden.^1^,^2^ Non-small cell lung cancer (NSCLC) accounts for the vast majority of lung cancer cases. For patients with advanced NSCLC, conventional chemotherapy offers limited efficacy and is associated with substantial toxicity.^3^ In recent years, immune checkpoint inhibitors (ICIs), particularly monoclonal antibodies targeting programmed cell death protein-1 (PD-1) and its ligand PD-L1, have achieved groundbreaking success in the treatment of advanced NSCLC.^4^ By blocking the PD-1/PD-L1 inhibitory signaling pathway, these agents restore T-cell function and reinvigorate antitumor immunity, thereby significantly improving survival outcomes in a subset of patients.^3^ Currently, PD-1/PD-L1 inhibitors as monotherapy are recommended as first-line treatment for patients with advanced NSCLC exhibiting high PD-L1 expression. Moreover, chemoimmunotherapy combinations have further overcome the limitations of PD-L1 expression and have become the new standard of care for patients with driver gene-negative disease.^5^ However, only 10-40% of patients derive durable benefit from ICI monotherapy in NSCLC.^6^ In addition, although immune-related adverse events occur less frequently than chemotherapy-associated toxicities, they may involve multiple organ systems and can be life-threatening in severe cases.^7^ Consequently, the development of combination strategies that can overcome immune resistance, expand the population of responders, enhance efficacy, and mitigate toxicity has become an urgent priority in current research. Thymalfasin (Thymosin α1) is a 28-amino acid peptide that promotes T-cell differentiation and proliferation, enhances dendritic cell antigen presentation, and upregulates tumor antigen expression. It has been approved in several countries for the treatment of immune deficiency-related conditions.^8^

A growing body of evidence indicates that thymalfasin exerts synergistic antitumor effects and protective activity against radio- and chemotherapy-induced toxicity in malignancies.^9^ Studies have demonstrated that thymalfasin can significantly inhibit the migratory and metastatic potential of NSCLC cell lines with varying levels of PD-L1 expression^10^ and improve five-years progression-free survival (PFS) and overall survival (OS) in patients with NSCLC.^11^ These findings suggest that thymalfasin modulates multiple critical steps in the ICI-mediated antitumor immune response cycle, thereby possessing the potential to synergize with ICIs while mitigating toxicity. However, robust data comparing the efficacy and safety of thymalfasin across different therapeutic regimens remain limited. Moreover, the impact of thymalfasin on dynamic changes in immune function and patient quality of life has not been adequately characterized. To address this gap, a retrospective cohort analysis was conducted to evaluate the efficacy and safety of thymalfasin in combination with various ICI-based regimens in patients with NSCLC in real-world clinical practice.

In addition, this study explored its potential effects on immune function, aiming to provide new insights for optimizing immunotherapy strategies in advanced NSCLC and maximizing patient benefit.

## METHODOLOGY

This study was designed as a retrospective cohort analysis. The sample size was determined by the total number of eligible patients who met the inclusion and exclusion criteria during the study period; no a priori sample size calculation was performed. One hundred and twenty patients with advanced(stage IIIB-IV) driver gene-negative NSCLC who were admitted to the Department of Oncology, Shijiazhuang People’s Hospital, between January 2021 to December 2024 were retrospectively included. Patients were divided into three Groups-According to the first-line treatment regimen they actually received: Group-A was administered platinum-based doublet chemotherapy, ICIs, and thymalfasin; Group-B was given single-agent chemotherapy, ICIs, and thymalfasin; Group-C received ICIs and thymalfasin.

### Ethical approval:

The study was approved by the Institutional Ethics Committee of Shijiazhuang People’s Hospital (No.:[2024]003; date: February 05, 2024), and written informed consent was obtained from all participants.

### Inclusion criteria:

- Age ≥18-65 years old; histopathological or cytological confirmation of NSCLC.
- Negative for major driver mutations(EGFR, ALK, ROS1, etc.).
- TNM(tumor, node, metastasis) stage IIIB-IV, not amenable to curative surgery or radiotherapy.
- Recipients of a first-line regimen based on ICIs.
- Treatment protocol included thymalfasin.
- At least one measurable lesion at baseline, with an Eastern Cooperative Oncology Group (ECOG) performance status score of 0-2.
- Availability of complete clinical records.
- Patients and their families who had good compliance with treatment and were willing and able to cooperate with the completion of this study.

### Exclusion criteria:

- Included the presence of other active malignancies.
- Severe active infection or uncontrolled autoimmune disease.
- Severe cardiac, hepatic, or renal dysfunction.
- Previous systemic antitumor therapy for advanced disease.
- Substantial missing data precluding efficacy or safety evaluation.
- Concurrent participation in another clinical trial that could potentially interfere with study outcomes.
- Patients with complicated asthma, pulmonary edema and other respiratory diseases.

### Treatment regimens:

ICIs: Patients received PD-1/PD-L1 inhibitors approved for the treatment of NSCLC, including pembrolizumab, sintilimab, camrelizumab, and tislelizumab. Administration protocols followed the respective package inserts and standard clinical practice. Chemotherapy agents: Platinum-based doublet chemotherapy (Group-A): a platinum compound (cisplatin or carboplatin) combined with one chemotherapy agent (e.g., pemetrexed, paclitaxel, nab-paclitaxel, or gemcitabine), administered at standard doses and cycles. Single-agent chemotherapy (Group-B): agents such as pemetrexed, gemcitabine, or docetaxel, administered at standard doses and cycles. Thymalfasin: Administered according to standard clinical practice and prescribing information, typically 1.6 mg subcutaneously twice weekly, with continuous use during treatment. Actual dosage and duration were based on medical records. Control Group-Considerations: This study primarily focused on comparing three thymalfasin- and ICI-based therapeutic strategies (Groups-A, B, and C). In accordance with the study design, no additional comparator arms (*e.g*., ICIs alone or ICIs + chemotherapy without thymalfasin) were established. All treatments were performed by the same group of doctors, which has been described in the text.

Clinical data were retrieved from the hospital electronic medical record system, including baseline characteristics and treatment information. Baseline characteristics refer to demographic information(age, sex), smoking history, pathological type (adenocarcinoma/squamous cell carcinoma/others), TNM stage, ECOG performance status, baseline laboratory tests (complete blood count, comprehensive metabolic panel), baseline PD-L1 expression level (tumor proportion score, if available), and baseline imaging data (computed tomography/magnetic resonance imaging). Treatment information includes specific ICIs administered, chemotherapy regimen and dosage, thymalfasin dosage, frequency, and total duration, treatment initiation date, number of treatment cycles, and reasons for treatment discontinuation (disease progression, toxicity, patient preference, etc.).

### Outcome measures:

Efficacy assessment: Tumor response was evaluated according to the criteria established by the Union for International Cancer Control and the WHO.^9^ Treatment responses were categorized as follows:

- Complete response (CR): Disappearance of all tumor lesions sustained for at least one month;
- Partial response (PR): ≥50% reduction in tumor burden without disease progression, sustained for at least one month, with no new lesions;
- Stable disease (SD): <50% reduction or ≤25% increase in tumor burden, sustained for at least one month;

Progressive disease (PD): >25% increase in tumor burden or the appearance of new lesions. Objective response rate (ORR) was calculated as (CR + PR cases) / total cases × 100%. Disease control rate (DCR) was calculated as (CR + PR + SD cases) / total cases × 100%. PFS was defined as the time from treatment initiation to the first documented disease progression. OS was defined as the time from treatment initiation to death or last follow-up.

### Safety assessment:

Adverse events were graded according to the National Cancer Institute Common Toxicity Criteria version 3.0 (NCI-CTC 3.0).

### Immunological function:

Peripheral venous blood (3 mL, fasting) was collected before treatment and at 6 months after treatment. Following centrifugation (4000 rpm, 5 cm radius, 10 min), plasma was harvested. Flow cytometry was used to quantify T-lymphocyte subsets, including CD3^+^, CD4^+^, and the CD4^+^/CD8^+^ ratio. Immunoglobulins M (IgM), G (IgG), and A (IgA) were measured using immunoturbidimetry.

### Quality of life:

The Chinese version of the Functional Assessment of Cancer Therapy-Lung (FACT-L) questionnaire was used to assess patients’ quality of life before and after treatment.^11^ The scale comprises 37 items across two domains, with lower scores indicating poorer quality of life. Exercise tolerance was assessed using the 6-minutes’ walk distance (6MWD) test.

### Statistical analysis:

All statistical analyses were performed using SPSS version 26.0. Continuous variables with a normal distribution were expressed as mean ± standard deviation (*x̅*±*s*), whereas those with a non-normal distribution were presented as median and interquartile range (M [P25, P75]). For normally distributed data with homogeneity of variance, one-way analysis of variance was applied, followed by pairwise comparisons using the least significant difference test. For non-normally distributed data, the Kruskal-Wallis H test was used, with pairwise comparisons conducted using the Mann-Whitney U test. The confidence interval was 95%. Categorical variables were summarized as counts and percentages(*n*, %), with comparisons performed using the chi-square (χ²) test or Fisher’s exact test as appropriate. PFS and OS were estimated using the Kaplan-Meier method, and survival differences between groups were assessed with the log-rank test. A *P*-value <0.05 was considered statistically significant.

## RESULTS

A total of 160 patients with advanced driver gene-negative NSCLC admitted to Shijiazhuang People’s Hospital were screened. Based on the inclusion and exclusion criteria, 40 patients were excluded (including 12 with driver gene-positive mutations, 10 with prior systemic therapy for advanced disease, 10 with incomplete records, three with concurrent active malignancies, and five with severe organ dysfunction). Ultimately, 120 eligible patients were enrolled in this study, including 42 in Group-A, 44 in Group-B, and 34 in Group-C. Baseline characteristics were comparable across the three groups, with no statistically significant differences(all *P>* 0.05), indicating good Group-Comparability Table-I.

**Table-I:** Baseline characteristics of patients in the three groups.

Item	Group-A (n = 42)	Group-B (n = 44)	Group-C (n = 34)	F/χ²-value	P-value
Age (years)	56.30±7.11	57.33±7.65	57.33±7.65	0.962	0.385
Sex (male/female, n)	25/17	24/20	20/14	0.252	0.882
TNM stage				3.635	0.458
IIIB	15	14	14		
IIIC	13	14	14		
IV	14	16	6		
Pathological type				1.234	0.873
Adenocarcinoma	18	17	16		
Squamous carcinoma	16	20	14		
Others	8	7	4		
ECOG score	0.98±0.56	1.02±0.59	1.00±0.70	0.062	0.940
PD-L1 expression				1.122	0.891
<1%	15	18	14		
1-49%	18	20	15		
≥50%	9	6	5		

**Fig.1:**
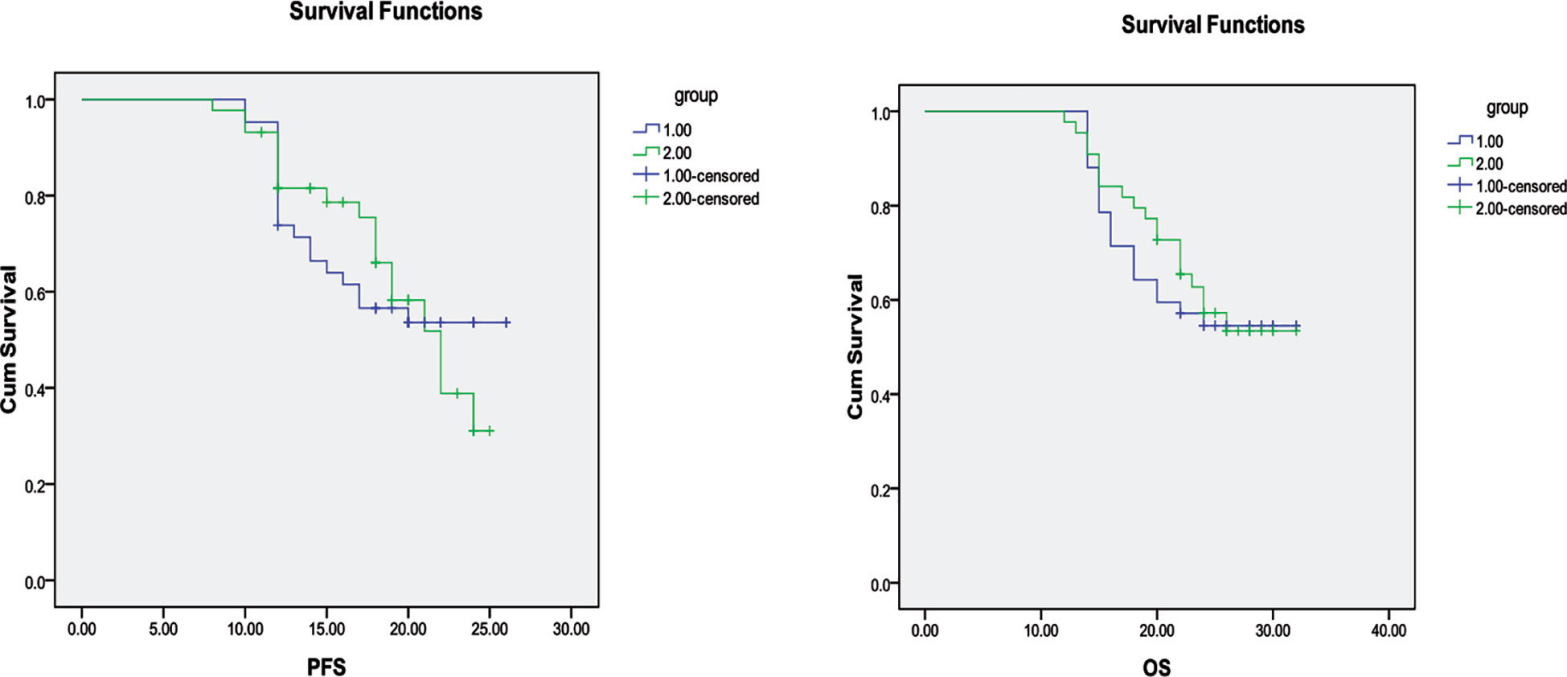
Comparison of PFS and OS between Groups-A and B.

**Fig.2:**
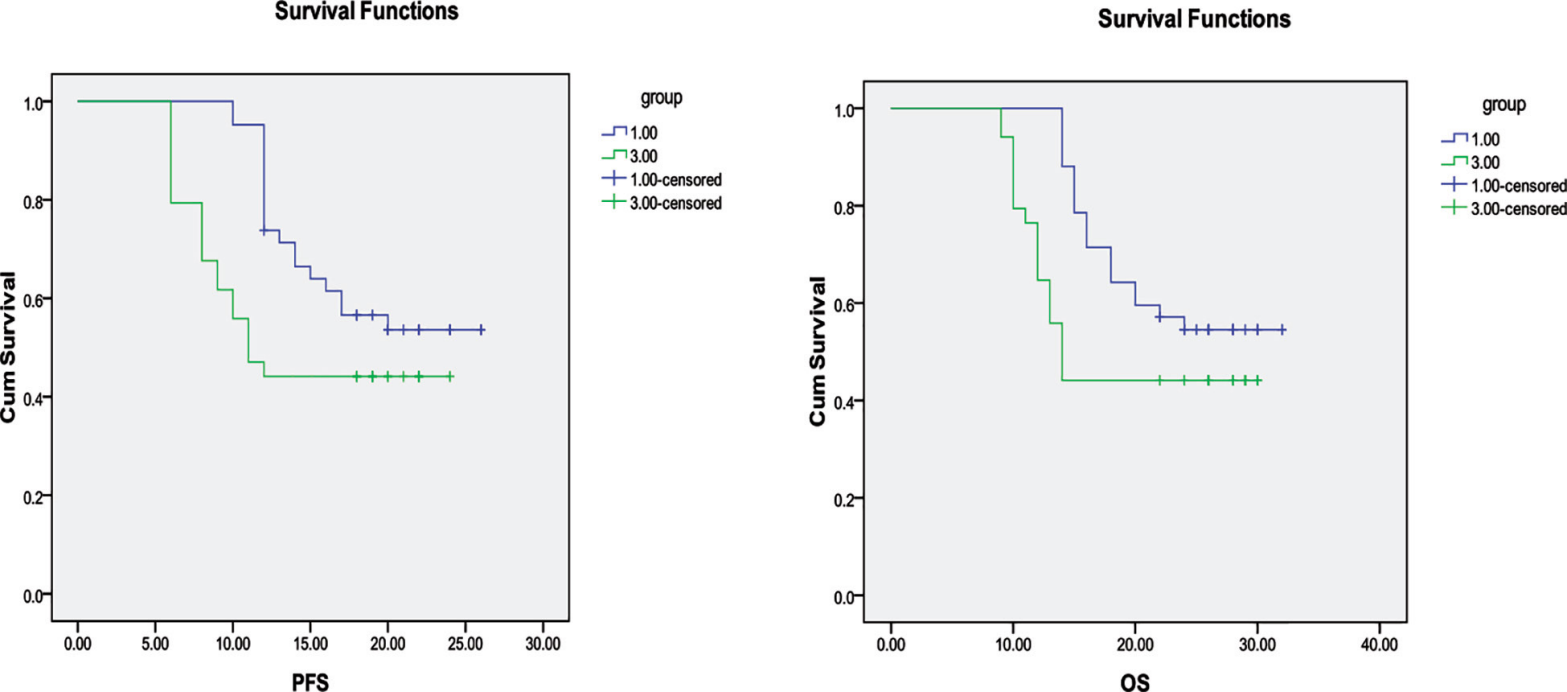
Comparison of PFS and OS between Groups-A and C.

After treatment, no CR cases were observed in any group. The number of patients achieving PR was 5(11.90%) in Group-A, 4(9.09%) in Group-B, and 2(5.88%) in Group-C. SD was observed in 35 patients(83.34%) in Group-A, 37(84.09%) in Group-B, and 28 (82.35%) in Group-C. There were no statistically significant differences in ORR or DCR among the three groups (all *P>* 0.05). Table-II. Median PFS and OS did not differ significantly between Groups-A and B (*P>* 0.05, respectively). However, both Groups-A and B demonstrated significantly longer median PFS and OS compared with Group-C (*P<* 0.05, respectively) Fig.1, 2, and 3. During treatment, adverse events were observed in all three groups. The incidence of bone marrow suppression, gastrointestinal reactions, fatigue, hepatic/renal impairment, and rash/alopecia did not differ significantly among the three groups (all *P>* 0.05) Table-III.

**Table-II:** Comparison of clinical efficacy among the three treatment regimens (n[%]).

Group	n	CR	PR	SD	PD	ORR	DCR
Group-A	42	0(0.00)	5(11.90)	35(83.34)	2(4.76)	5(11.90)	40(95.24)
Group-B	44	0(0.00)	4(9.09)	37(84.09)	3(6.82)	4(9.09)	41(93.18)
Group-C	34	0(0.00)	2(5.88)	28(82.35)	4(11.77)	2(5.88)	30(88.23)
χ² value		2.022				0.819	1.375
P-value		0.732				0.664	0503

**Fig.3:**
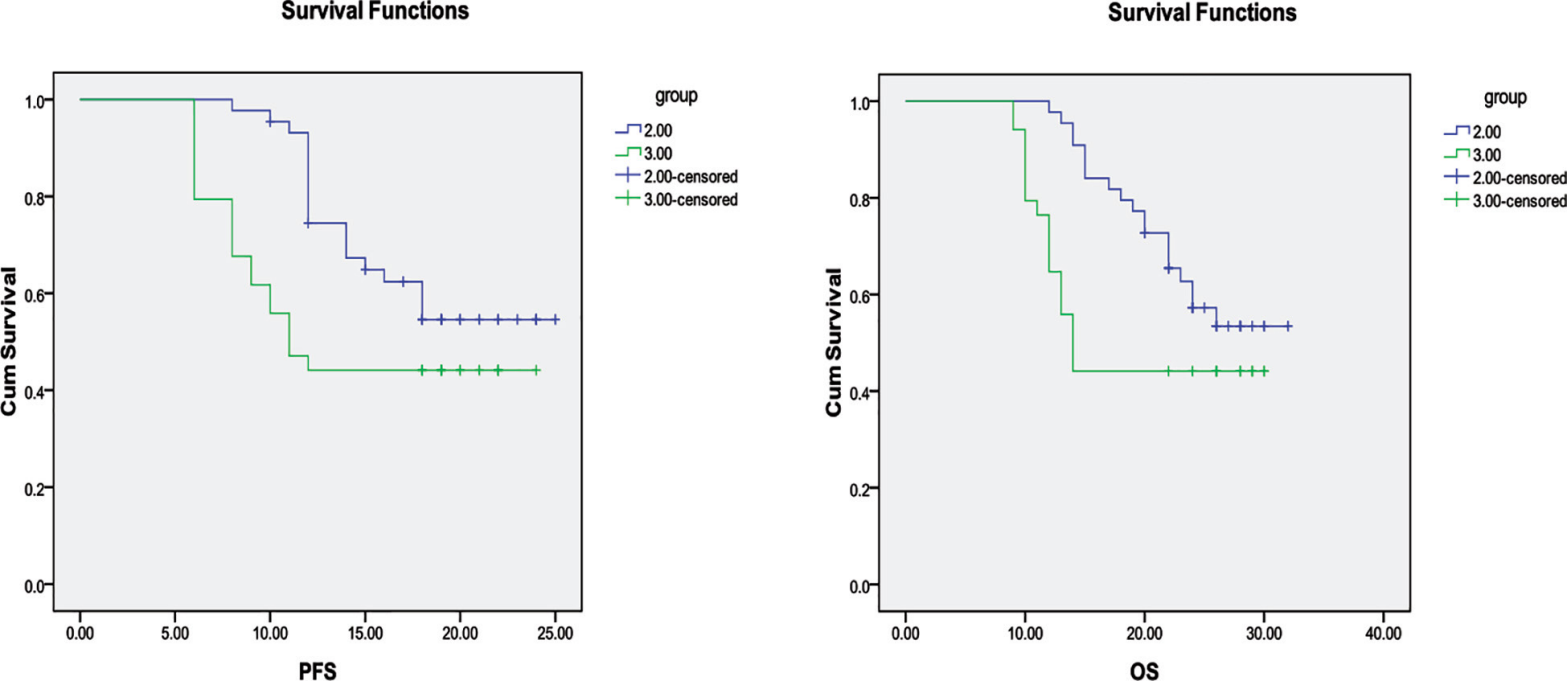
Comparison of PFS and OS between Groups-B and C.

**Table-III:** Comparison of adverse events among the three groups (n[%]).

Group	n	Bone marrow suppression	Gastrointestinal reactions	Fatigue	Hepatic/Renal impairment	Rash/alopecia
Group-A	42	14(33.33)	17(40.48)	8(19.05)	16(38.10)	9(21.43)
Group-B	44	15(34.09)	14(31.82)	6(13.64)	13(29.55)	6(13.64)
Group-C	34	11(32.35)	12(35.29)	5(14.71)	12(35.29)	4(11.76)
*χ* ^2^		0.026	0.707	0.517	0.725	1.568
P-value		0.987	0.702	0.772	0.696	0.456

Among the Three Groups-Before treatment, there were no significant differences among the three groups in CD3^+^, CD4^+^, CD4^+^/CD8^+^, IgM, IgG, or IgA levels (all *P>* 0.05). After treatment, all three groups showed significant improvements in these immune parameters compared with baseline (all *P <* 0.05). Post-treatment comparisons revealed the following: No significant difference in CD3^+^ was observed between Groups-A and B (*P>* 0.05), but both Groups-A and B had significantly higher CD3^+^ levels than Group-C (both *P<* 0.05). Significant differences in CD4^+^ levels were found among all pairwise comparisons (*P<* 0.05, respectively). No difference in CD4^+^/CD8^+^ was noted between Groups-B and C (*P>* 0.05), while Groups-A *vs*. B and A *vs*. C showed significant differences (*P<* 0.05). There were no significant differences in IgM levels among the three groups (*P>* 0.05). IgG levels did not differ greatly between Groups-B and C (*P>* 0.05), but Groups-A *vs*. B and A *vs*. C were significantly different (*P<* 0.05, respectively). IgA levels were significantly different among all pairwise comparisons (*P<* 0.05, respectively) Table-IV.

**Table-IV:** Comparison of immune function among the three Groups-Before and after treatment (*x̅*±*s*).

Outcome Measures	Time Point	Group-A	Group-B	Group-C	F-value	P-value
CD3+ (%)	Pre-treatment	42.62±6.27	44.19±5.47	42.12±6.85	1.241	0.293
	Post-treatment	48.30±6.34	46.93±6.14	42.66±6.78	7.766	<0.001
CD4+ (%)	Pre-treatment	27.33±4.04	27.28±4.43	26.44±3.55	0.554	0.576
	Post-treatment*	38.00±4.54	35.67±4.66	33.08±5.26	9.889	<0.001
CD4+/CD8+ (%)	Pre-treatment	1.28±0.10	1.28±0.07	1.28±0.07	0.051	0.950
	Post-treatment*	1.74±0.18	1.64±0.19	1.57±0.18	8.987	<0.001
IgM (g/L)	Pre-treatment	1.41±0.65	1.43±0.64	1.31±0.70	0.339	0.713
	Post-treatment*	2.74±0.63	2.50±0.59	2.58±0.63	1.630	0.200
IgG (g/L)	Pre-treatment	1.36±0.29	1.31±0.33	1.37±0.30	0.422	0.657
	Post-treatment*	2.85±0.74	2.47±0.66	2.28±0.47	7.858	0.001
IgA (g/L)	Pre-treatment	1.36±0.29	1.31±0.33	1.36±0.29	0.340	0.713
	Post-treatment*	2.83±0.74	2.44±0.65	2.16±0.36	11.497	<0.001

Before treatment, no significant differences were observed among the three groups in FACT-L or 6MWD scores (all P> 0.05). After treatment, FACT-L and 6MWD scores significantly improved in all groups compared with baseline (all P< 0.05). Pairwise comparisons showed significant differences in FACT-L scores among all three groups (all P< 0.05). No significant difference was observed in 6MWD scores between Groups-B and C(P> 0.05), while Groups-A vs. B and A vs. C both showed significant differences (both P< 0.05) Table-V.

**Table-V:** Comparison of quality of life and exercise tolerance among the three groups (*x̅*±*s*).

Group	FACT-L score (points)		6MWD (m)	
	Pre-treatment	Post-treatment	Pre-treatment	Post-treatment
Group-A	76.98±6.75	89.52±6.46	383.17±8.45	346.67±6.78
Group-B	78.45±7.36	88.45±7.34	384.23±8.92	352.61±8.81
Group-C	75.38±6.47	82.00±6.23	380.44±8.69	355.21±6.76
F-value	1.904	11.755	1.882	12.991
P-value	0.154	<0.001	0.157	<0.001

## DISCUSSION

This retrospective cohort study evaluated the efficacy, safety, and immunological function of thymalfasin combined with three different ICI-based regimens in patients with advanced driver gene-negative NSCLC. The results demonstrated no statistically significant differences among the three groups in ORR and DCR, and no CR cases were observed, suggesting that thymalfasin did not markedly enhance short-term tumor regression. However, significant differences in survival outcomes were identified. Specifically, both chemotherapy-containing regimens (Groups-A and B) achieved superior PFS and OS compared with ICI plus thymalfasin alone (Group-C). These findings are highly consistent with current first-line treatment evidence for advanced NSCLC.^12^ Notably, thymalfasin was included in all treatment arms, indicating that its primary contribution may be to reinforce immune activation and/or maintain immune homeostasis on the basis of already effective regimens, rather than serving as an independent determinant of survival benefit.^13^ Thymalfasin exerts its immunomodulatory effects by promoting T-cell differentiation and proliferation and enhancing dendritic cell function, thereby acting on multiple stages of the cancer-immunity cycle.^14^ In this study, thymalfasin may have optimized the immune microenvironment established by ICIs, particularly in combination with chemotherapy (Groups-A and B), where immunogenic cell death and immune activation were already effectively induced. Under these conditions, its synergistic potential was more fully realized, echoing previous reports that thymalfasin can improve long-term survival in patients with NSCLC.

From a safety perspective, the incidence of adverse events did not differ significantly among the three groups. This finding carries important clinical implications, as it confirms that thymalfasin did not substantially increase treatment-related toxicities when combined with ICIs, regardless of whether chemotherapy was included. Of particular note, in the theoretically more toxic platinum-based doublet chemotherapy group (Group-A), the addition of thymalfasin did not result in a higher rate of adverse events. This observation supports the hypothesis that thymalfasin may possess toxicity-mitigating potential, possibly by improving immune function or exerting tissue-protective effects, thereby enabling patients to better tolerate more intensive treatment regimens. This aligns with previous reports describing its protective role during radiotherapy and chemotherapy.^14^

This study also analyzed the dynamic changes in immune function before and after treatment. Post-treatment, both cellular and humoral immune parameters improved significantly compared with baseline across all three groups, demonstrating that thymalfasin combined with ICIs can systematically enhance immune competence in patients. Notably, the extent of immune improvement appeared to be regimen-dependent. Group-A exhibited the most pronounced enhancements in key parameters, particularly CD4^+^ T-cell counts as well as IgG and IgA levels, which were significantly superior to those in Group-C and partially better than those in Group-B. These findings provide a biological rationale for the superior survival benefit and improved quality of life observed in Group-A. The synergistic mechanism may involve chemotherapy-induced immunogenic cell death releasing abundant tumor antigens, ICIs reversing PD-1/PD-L1-mediated T-cell suppression,^15^ and thymalfasin enhancing T-cell function, antigen presentation, and antibody production at multiple checkpoints. Together, these processes may establish a more robust and durable antitumor immune response.^16^ Importantly, such detailed monitoring of immune function dynamics addresses a common gap in similar efficacy-focused studies, which often lack mechanistic exploration. Our results corroborate findings from prior domestic and international studies and further highlight how different therapeutic modalities can variably influence the degree of immune enhancement.

Regarding patient-reported outcomes, FACT-L scores improved significantly across all Groups-After treatment, with the greatest improvement observed in Group-A, which was markedly superior to Group-C. This pattern parallels the survival benefits and immune function enhancements most pronounced in Group-A. Particularly noteworthy were the results for exercise tolerance. Although post-treatment 6MWD decreased relative to baseline in all groups, reflecting the overall burden of anticancer therapy, the decline was significantly smaller in Group-A (platinum-based doublet regimen) compared with Groups-B and C. Given that Group-A received the regimen theoretically most detrimental to physical performance, this finding strongly suggests that the combination of thymalfasin with platinum-based chemotherapy and ICIs may confer a unique advantage in preserving functional capacity. This benefit is likely attributable to thymalfasin’s immunomodulatory effects in reducing systemic inflammation, improving overall health status, and exerting potential tissue-protective actions, thereby offsetting part of chemotherapy’s negative impact on physical endurance and ultimately enhancing both treatment tolerability and quality of life.^17^

To our knowledge, this study is the first to systematically compare thymalfasin across three mainstream ICI-based regimens (intensified chemotherapy + ICI, attenuated chemotherapy + ICI, and ICI monotherapy) in a real-world clinical setting, thereby providing a nuanced perspective on its potentiating properties. The results corroborate preclinical studies and prior clinical observations suggesting synergism between thymalfasin and ICI/chemotherapy^18^ and particularly highlight that such synergy may yield the most comprehensive benefit when integrated into the current standard of care, platinum-based doublet chemotherapy plus ICI.^19^,^20^ From a safety perspective, this study, with a relatively large sample size(*n =* 120) and across diverse treatment modalities, confirmed that the addition of thymalfasin did not significantly increase treatment-related toxicity. This reinforces confidence in its clinical application and provides an important extension to domestic and international reports consistently attesting to its favorable safety profile.

### Limitations:

First, as a retrospective analysis, it is inherently subject to risks of selection and information bias. Treatment allocation (*i.e*., assignment to Groups-A, B, or C) was non-randomized and may have been influenced by unmeasured confounding factors such as physician judgment, patient performance status, comorbidities, or financial considerations. Second, the relatively limited sample size and single-center design may constrain the generalizability of the findings and reduce the statistical power for subgroup-analyses. Moreover, heterogeneity in the types of ICIs used could not be fully explored through stratified analysis.

## CONCLUSIONS

This study demonstrates that thymalfasin combined with ICI-based regimens in the treatment of advanced driver gene-negative NSCLC is well tolerated. This treatment regimen can systematically improve immune function and enhance quality of life. These findings provide supportive evidence for the integration of thymalfasin into immunotherapy-based treatment strategies for advanced NSCLC.

### Authors’ Contributions:

**PW:** Literature search, study design and manuscript writing.

**XZ** and **CX:** Data collection, data analysis and interpretation. Critical Review.

**GL** and **YZ:** Critical Review, manuscript revision, validation and is responsible for the integrity of the study. All authors have read and approved the final manuscript.
